# Wearable sensors objectively measure gait parameters in Parkinson’s disease

**DOI:** 10.1371/journal.pone.0183989

**Published:** 2017-10-11

**Authors:** Johannes C. M. Schlachetzki, Jens Barth, Franz Marxreiter, Julia Gossler, Zacharias Kohl, Samuel Reinfelder, Heiko Gassner, Kamiar Aminian, Bjoern M. Eskofier, Jürgen Winkler, Jochen Klucken

**Affiliations:** 1 Department of Molecular Neurology, University Hospital Erlangen, Friedrich-Alexander Universität Erlangen-Nürnberg (FAU), Erlangen, Germany; 2 Digital Sports Group, Pattern Recognition Lab, Department of Computer Science, FAU Erlangen-Nürnberg, Erlangen, Germany; 3 ASTRUM IT GmbH, Am Wolfsmantel 2, Erlangen, Germany; 4 Ecole Polytechnique Fédérale de Lausanne (EPFL), Laboratory of Movement Analysis and Measurement, Station 11, Lausanne, Switzerland; Oslo Universitetssykehus, NORWAY

## Abstract

Distinct gait characteristics like *short steps* and *shuffling gait* are prototypical signs commonly observed in Parkinson’s disease. Routinely assessed by observation through clinicians, gait is rated as part of categorical clinical scores. There is an increasing need to provide quantitative measurements of gait, e.g. to provide detailed information about disease progression. Recently, we developed a wearable sensor-based gait analysis system as diagnostic tool that objectively assesses gait parameter in Parkinson’s disease without the need of having a specialized gait laboratory. This system consists of inertial sensor units attached laterally to both shoes. The computed target of measures are spatiotemporal gait parameters including stride length and time, stance phase time, heel-strike and toe-off angle, toe clearance, and inter-stride variation from gait sequences. To translate this prototype into medical care, we conducted a cross-sectional study including 190 Parkinson’s disease patients and 101 age-matched controls and measured gait characteristics during a 4x10 meter walk at the subjects’ preferred speed. To determine intraindividual changes in gait, we monitored the gait characteristics of 63 patients longitudinally. Cross-sectional analysis revealed distinct spatiotemporal gait parameter differences reflecting typical Parkinson’s disease gait characteristics including short steps, shuffling gait, and postural instability specific for different disease stages and levels of motor impairment. The longitudinal analysis revealed that gait parameters were sensitive to changes by mirroring the progressive nature of Parkinson’s disease and corresponded to physician ratings. Taken together, we successfully show that wearable sensor-based gait analysis reaches clinical applicability providing a high biomechanical resolution for gait impairment in Parkinson’s disease. These data demonstrate the feasibility and applicability of objective wearable sensor-based gait measurement in Parkinson’s disease reaching high technological readiness levels for both, large scale clinical studies and individual patient care.

## Introduction

Gait is an important indicator for quality of life [1] and is examined during the clinical diagnostic workup of Parkinson’s disease (PD). Introduced in the 1980s to allow a standardized clinical examination, the motor part of the Unified Parkinson’s Disease Rating Scale (UPDRS-III) provides the physician with a valuable standardized clinical examination to rate motor impairment in PD. UPDRS-III includes several gait-associated items, e.g., rigidity and agility of the lower extremities. However, the evaluation of gait in PD patients uses a single item categorized from “normal,” to “walks slowly, may shuffle with short steps,” “walks with difficulty, little or no assistance, some festination, short steps or propulsion,” and “severe disturbance, frequent assistance,” to “cannot walk” [2]. Although the UPDRS-III assists the physician to rate general motor impairment in PD, quantitative information on gait impairment is very limited. For example, to determine whether somebody shuffles with their feet, i.e. not lifting the feet fully from the ground, is very subjective. Therefore, being able to implement an objective assessment of gait parameters by mobile means could enable quantification of distinct PD gait patterns, support individual monitoring of disease progression, and help to evaluate response to treatment.

**Fig 1:**
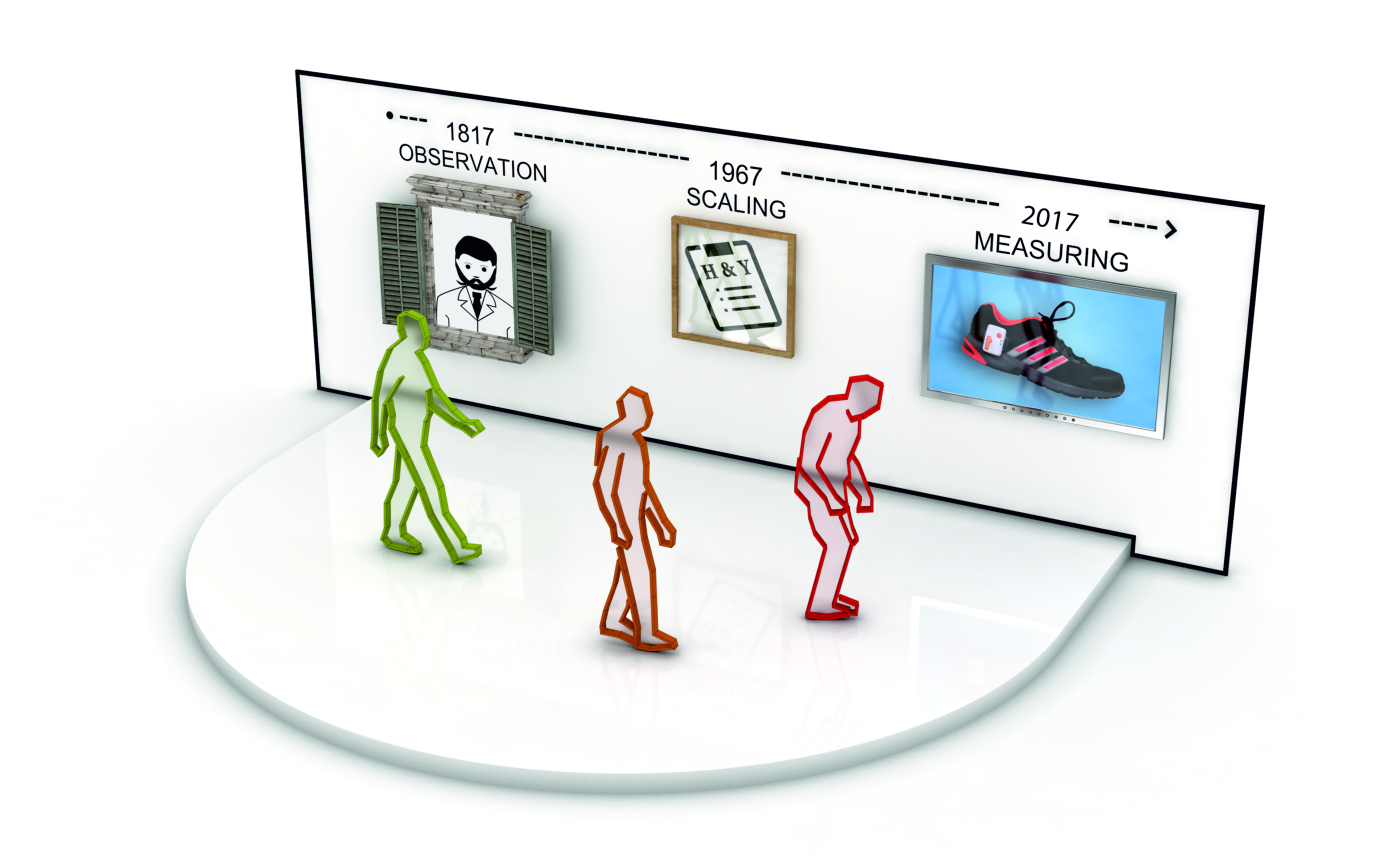
Observation, rating, and measuring gait in PD. Gait is commonly assessed by observation. The clinician uses a standardized scale, e.g., the motor part of the UPDRS, to rate gait performance. Nowadays, body worn sensors attached to the shoe enable measurement of prototypical PD gait features.

Although instrumented motion analysis systems have been used for several decades, utility of such sensing technology has been mainly restricted to an experimental movement laboratory environment. Video-based motion-capture systems or instrumented walkway systems are considered to be the gold standard for capturing gait parameters, but are costly and require specialized movement laboratories [3, 4]. At present, wearable body-sensor network technology is moving into the routine clinical workup, complementing advanced movement assessment with stationary and complex kinematographic analysis [5–7] (Fig 1). These wearable motion sensor systems are increasingly well suited for gait analysis during everyday life, offering a means to objectively deliver individualized gait signatures [8–10]. However, albeit the general agreement on the need for quantitative assessment of PD, there is a lack of studies validating the use of wearable sensor technology in clinical assessment of PD [11].

Mobile inertial measurement units consisting of accelerometers and gyroscopes are able to objectively track motion throughout gait [10, 12, 13]. Gait analysis in PD using body worn sensors attached to the lower limbs has been developed recently [14–17]. We previously demonstrated the feasibility of our wearable sensor-based gait system to distinguish PD patients from controls [17] and validated all algorithms for stride parameter calculation [18–21]. Specifically, as segmentation of gait signals into single steps is an important basis for objective gait analysis, we have previously validated our segmentation procedure in PD patients [18]. We recently assessed the concurrent validity and test-retest reliability of the body worn sensor-based gait analysis system in PD patients [22]. The wearable sensor-based gait system accurately captured gait parameters as compared to the reference camera-based system.

The aim of this study was to show that wearable sensor technology assesses spatiotemporal gait parameters (stride length, stride time, gait velocity, stride and stance phase times, foot clearance, heel-strike (HS) and toe-off (TO) angles and gait variation measures) in a large cohort of PD patients to determine both group differences and individual changes over time. In a cross-sectional study design, we quantified gait parameters in 190 PD patients at different disease stages and compared them to 101 age-matched controls. To monitor individualized gait parameter changes during the course of the disease, we longitudinally compared the gait parameters of 63 PD patients.

## Methods

### Study population

Written informed consent was obtained from all participants (IRB-approval-Re. -No. 4208, 21.04.2010, IRB, Medical Faculty, Friedrich-Alexander University Erlangen-Nürnberg, Germany). Patients diagnosed with PD according to the consensus criteria of the German Society of Neurology analogue to the National Institute of Neurological Disorders and Stroke (NINDS) diagnostic criteria for PD were recruited during their regular visit in the movement disorder outpatient center at the University Hospital Erlangen from May 3^rd,^ 2010 until July 31^st,^ 2014. PD patients were staged according to Hoehn and Yahr (H&Y). Clinical evaluation according to the UPDRS-III rating was obtained immediately prior to gait assessment (< 30 min) to reduce the influence of motor fluctuations [2]. The gait tests were performed in the patients’ “ON” motor state. Patients had to be able to walk independently (H&Y<4, UPDRS gait item <3). Additional exclusion criteria consisted of non-PD related gait impairments (e.g., spinal or musculo-skeletal diseases), atypical Parkinson syndromes, spasticity, stroke, neuropathy, myelopathy, hydrocephalus, and severe cognitive impairment. To obtain quantitative gait data from controls, we recruited 101 age-matched controls with no signs of PD and/or other motor impairments as defined by the exclusion criteria mentioned above. With respect to age, height, weight, and body-mass-index (BMI), PD and control cohorts were comparable (Table 1). PD patients reached significantly higher scores for depressive symptoms as assessed by the Zung Self-Rating Depression Scale (SDS) than controls (independent t-test, p < 0.05) [23, 24].

**Table 1:** Clinical characteristics of PD patients and healthy controls.

	PD (N = 190)	Controls (N = 101)
Age (years)	63.7 ± 0.8 [36–85]	61.2 ± 1.1 [41–84]
Sex		
Male	125	45
Female	65	56
Height (m)	1.72 ± 0.01	1.70 ± 0.01
Male	1.76 ± 0.01	1.76 ± 0.01
Female	1.64 ± 0.01	1.65 ± 0.01
Weight (kg)	77.5 ± 1.1	75.7 ± 1.3
BMI	26.1 ± 0.3	26.1 ± 0.4
Zung	48.0 ± 10.8 (n = 178)*	37.3 ± 1.0 (n = 90)*
Disease duration (years)	5.5 ± 0.4 [0–21]	
Hoehn and Yahr stage	2.12 ± 0.06	
I	N = 63	
II	N = 65	
III	N = 62	
UPDRS—III total	18.6 ± 0.8 [2–50]	
Low (0–12)	N = 68	
Middle (13–22)	N = 58	
High (>22)	N = 64	
Gait item		
0	N = 71	
1	N = 94	
2	N = 25	
Postural Stability item		
0	N = 90	
1	N = 81	
2	N = 19	

Values presented as mean ± SEM. Range is given in square brackets. Significance was determined by *t-test*.

* indicates P < 0.05

The following sub-items of the UPDRS-III that are associated with lower extremity function (UPDRS-III-LE) were selected for sub-analysis: bradykinesia, rigidity of neck and lower extremities, tremor of lower extremities, leg agility, posture, gait, arising from chair, and postural stability. PD patients were categorized based on the level of motor impairment in three groups with low (UPDRS-III_low_: 1–12), middle (UPDRS-III_middle_: 13–22), and high (UPDRS-III_high_: ≥ 23) as published previously [17].

Participants, walked freely at a comfortable, self-chosen speed in an obstacle free and flat environment for 4x10 meter. After each 10-meter walk, participants were instructed to turn 180 degrees. Due to the stride segmentation algorithm, turning strides were not analyzed. The first turn was clockwise, the second counter-clockwise, and the third again clockwise.

For the retrospective longitudinal analysis of the study, we retrospectively evaluated 63 PD patients who performed the 4x10 meter walking test at baseline and for a second time during a regular follow-up visit. Time between the baseline and second visit varied between 1 and 46 months. All 63 PD patients were grouped according to the clinician’s rating of the UPDRS-III item “gait” into three categories: stable (S; no changes in gait item rating), improved (I; increase in gait item rating) or worsened (W: drop in gait item rating).

### Sensor platform and stride parameter calculations

Data were collected with an inertial sensor measurement system consisting of two sensor units (Shimmer Sensing, Dublin, Ireland), including (1) a tri-axial accelerometer (Freescale Semiconductors MMA7361, range ±6 g, sensitivity of 200mV/g) and a (2) tri-axial gyroscope (InvenSense 500 series, range ± 500°/sec, sensitivity ± 2 mV/°/sec). The sensor units were attached to the lateral side of each shoe (S1A Fig). Data were streamed wirelessly via Bluetooth^®^ with a sampling rate of 51.2 Hz. The following gait parameters were extracted from each stride: stride length, stride time, gait velocity, cadence, stance phase time, swing phase time, HS angle, TO angle, gait variation, and foot clearance [15, 19, 20] (S1 Table and S1B Fig). Stride length is either given normalized or non-normalized to height [25, 26].

A robust, template-based stride detection algorithm automatically identified and segmented strides from gait tests, this way excluding turning strides for the calculation of gait parameters as previously reported [18, 27]. Briefly, strides were segmented using a multi-dimensional dynamic time warping and normalized to identify specific patterns, e.g. a stride, in a continuous sensor signal (S2 Fig). The segmented strides were then used to detect gait events (midstance, HS, and TO). To determine TO and HS angles, the angle was estimated from the accelerometer signal on midstance and the angle course during one stride was calculated from the gyroscope signals. At midstance, we used a zero-velocity update algorithm [28]. We integrated the acceleration signal from two consecutive midstances, depending on the angle during one stride, to estimate distance and stride length, as well as height of the toe and maximum toe clearance. To calculate temporal stride parameters, we measured the time from HS to HS, HS to TO, and TO to HS.

The algorithms for the spatial and temporal stride parameters using our sensor setup and location have been previously validated [18–20]. A previously published algorithm for the calculation of foot clearance was adapted and modified to our sensor setup to calculate foot clearance without manual shoe size input [15, 20].

### Statistical analysis

For demographic and clinical characteristics, independent *t* tests were used to compare baseline values between PD and control groups. To detect differences in the sensor-measured gait parameters of PD patients and controls in the cross-sectional study, we applied one-way ANOVA followed by Bonferroni’s post-hoc test. To detect changes during longitudinal monitoring of individual gait parameters, we also used one-way ANOVA followed by Bonferroni’s post-hoc test.

Data are expressed as mean values ± SEM. The significance level was set at p < 0.05. Statistical analysis was performed using the IBM SPSS software version 24 (IBM Deutschland GmbH, Germany). Figures were configured using PRISM 6.0 (GraphPad Software, San Diego, USA), PowerPoint (Microsoft, Redmond, USA), and CorelDraw X6 (Corel Corporation, Ottawa, Canada).

## Results

### Wearable sensor-based gait analysis detects “short steps” and “shuffling gait”

First, by choosing a cross-sectional approach of 190 PD patients and 101 age-matched controls, we observed significant differences between both groups in all sensor-measured spatiotemporal gait parameters (Table 2). Shuffling was mirrored best by reduced foot clearance as well as altered HS and TO angles. Foot clearance in controls averaged 14.3 ± 0.5 cm, whereas in PD patients foot clearance was significantly reduced by ∼ 24% to 10.6 ± 0.3 cm. The lifting of the foot reflected by the HS angle was robustly reduced in PD patients by ∼21.5%, whereas the TO angle did not significantly differ between the groups. Normalized stride length was significantly reduced by ∼ 7% in the PD cohort, whereas normalized stride time significantly increased by ∼ 5%. This pattern was associated with a significant decrease in cadence and gait velocity, reflecting typical bradykinetic features of gait in PD. Since the initiation strides profoundly alter the averaged gait parameters, we analyzed the distinct gait parameters without the first two initiation strides and confirmed these results (S2 Table).

**Table 2:** Gait parameters of the 4x10 meter walk *with* initiation steps.

Variables	PD	Controls	P
**Stride length [m]**	1.15 ± 0.02	1.23 ± 0.02	**< 0**.**001**
**Norm. stride length**	0.66 ± 0.01	0.72 ± 0.01	**< 0.001**
**Stride time [s]**	1.1 ± 0.01	1.04 ± 0.01	**< 0**.**001**
**Gait velocity [m/s]**	1.06 ± 0.01	1.2 ± 0.02	**< 0**.**001**
**Cadence [spm]**	55.1 ± 0.3	58.4 ± 0.8	**< 0**.**001**
**Stance phase time [%]**	65.0 ± 0.2	63.9 ± 0.2	**< 0**.**001**
**Swing phase time [%]**	34.9 ± 0.2	35.9 ± 0.2	**< 0.001**
**Foot clearance [cm]**	10.6 ± 0.3	14.3 ± 0.5	**< 0**.**001**
**Heel-strike angle [°]**	19.0 ± 0.7	22.1 ± 1.0	**< 0**.**01**
**Toe-off angle [°]**	-54.8 ± 0.7	-56.4 ± 0.9	> 0.05

Values presented as mean ± SEM. Significance was determined by *t-test*.

Abbreviations: Norm. = normalized

### Short steps and shuffling gait in different PD stages

**Fig 2:**
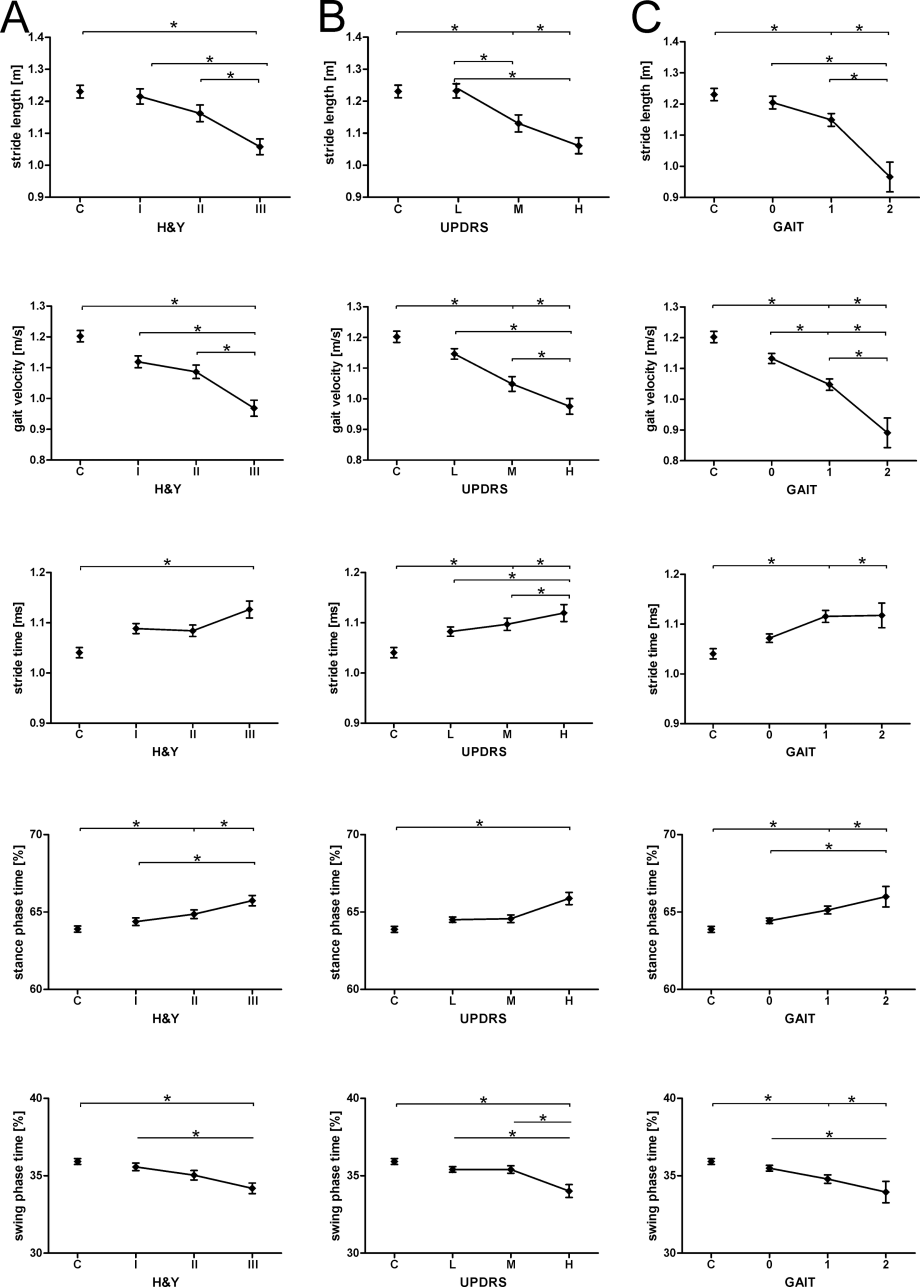
Measures of short steps in PD patients and controls (cross-sectional study). Stride length, gait velocity, stride time, stance phase, and swing phase time were calculated for controls and PD patients grouped according to H&Y disease stage (A), UPDRS-III total score (B), and the single item "gait" of the UPDRS-III (C). Group data are displayed as mean ± SEM. and compared using one-way ANOVA followed by Bonferroni’s post-hoc test. *p < 0.05.

As the disease progresses, gait impairment associated with reduced mobility becomes more prevalent and increasingly limits the quality of life of PD patients [1]. Therefore, we first compared gait pattern related to short steps between different disease stages according to H&Y. Overall, there was a significant difference in normalized stride length as well as to height, gait velocity, stride time, stance phase time, and swing phase time between PD and controls as disease progresses (Fig 2A). Patients in a moderate stage of the disease (H&Y stage 3) showed significantly reduced normalized stride length (0.62 ± 0.01) and gait velocity (0.97 ± 0.03 m/sec) compared to PD patients in their early stage (H&Y stage 1, 0.69 ± 0.01 normalized stride length and 1.12 ± 0.02 m/sec gait velocity, One-way ANOVA followed by Bonferroni’s post-hoc test, p < 0.05). In contrast, we did not detect differences in stride time between the different disease stages. Changes in stance phase time and stride phase time between H&Y stages 1 and 3 were modest (~1.3% and ~1.4%, respectively).

Next, we asked whether spatiotemporal gait parameters corresponded to motor impairment defined by the total UPDRS-III score (Fig 2B). All five spatiotemporal gait parameters were significantly impaired in PD patients with high motor impairment, i.e., UPDRS_high._ Stride length and gait velocity were also significantly reduced in UPDRS_high_ patients compared to low (UPDRS_low_) and medium motor impairment (UPDRS_middle_). According to the definition of the gait item of the UPDRS-III, a clinician rates the gait of PD patients by observing mainly changes in stride length. Therefore, we asked how wearable sensor-based gait parameters corresponded to the clinician’s gait rating. Specifically, we observed that stride length and gait velocity decreased, corresponding with the clinician’s rating of the UPDRS-III item gait. Stride time and stance phase time increased, also reflecting gait impairment (Fig 2C).

**Fig 3:**
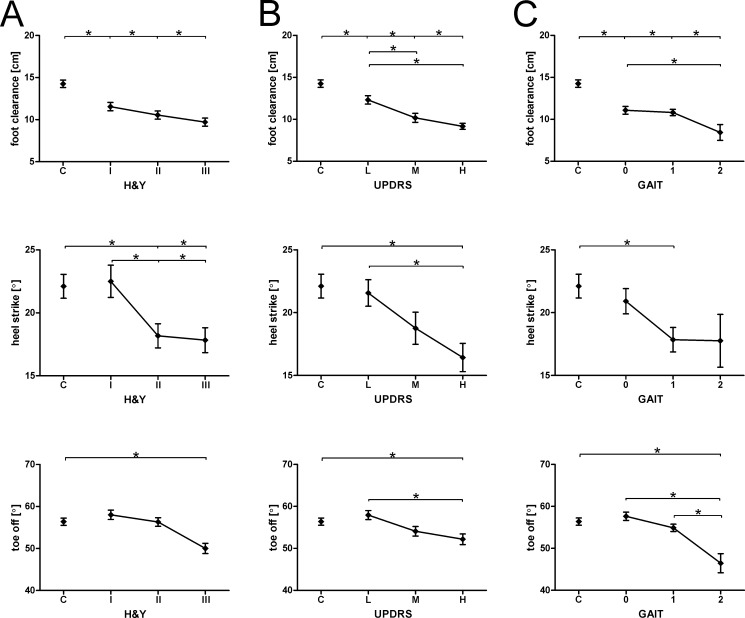
Measures of shuffling gait in PD patients and controls (cross-sectional study). Foot clearance as well as heel strike and toe off angles are depicted according to H&Y disease stage (A), UPDRS-III total score (B), and rating of the single item "gait" of the UPDRS-III (C). Group data are displayed as mean ± SEM. and compared using one-way ANOVA followed by Bonferroni’s post-hoc test. *p < 0.05.

The typical features of shuffling gait increased with the progression of disease. Foot clearance was significantly reduced and discriminated between PD patients at H&Y 1–3 and controls (Fig 3A). Foot clearance was already significantly decreased at the onset of disease (controls 14.3 ± 0.4 cm *vs*. H&Y 1 11.6 ± 0.5 cm). Foot clearance was also significantly reduced in all H&Y stages when patients were stratified according to the level of motor impairment (Fig 3B) and UPDRS-III gait item rating (Fig 3C). With advanced disease stages as well as motor and gait impairment, HS and TO angles were significantly reduced (Fig 3A–3C). Based on these findings, foot clearance was the most sensitive indicator of shuffling gait already being present early in the disease. Thus, we objectively measure and quantify both short steps and shuffling gait in PD, reflecting the progression of disease stages, levels of impairment of motor function and gait.

### Gait variation in PD is associated with postural instability

**Fig 4:**
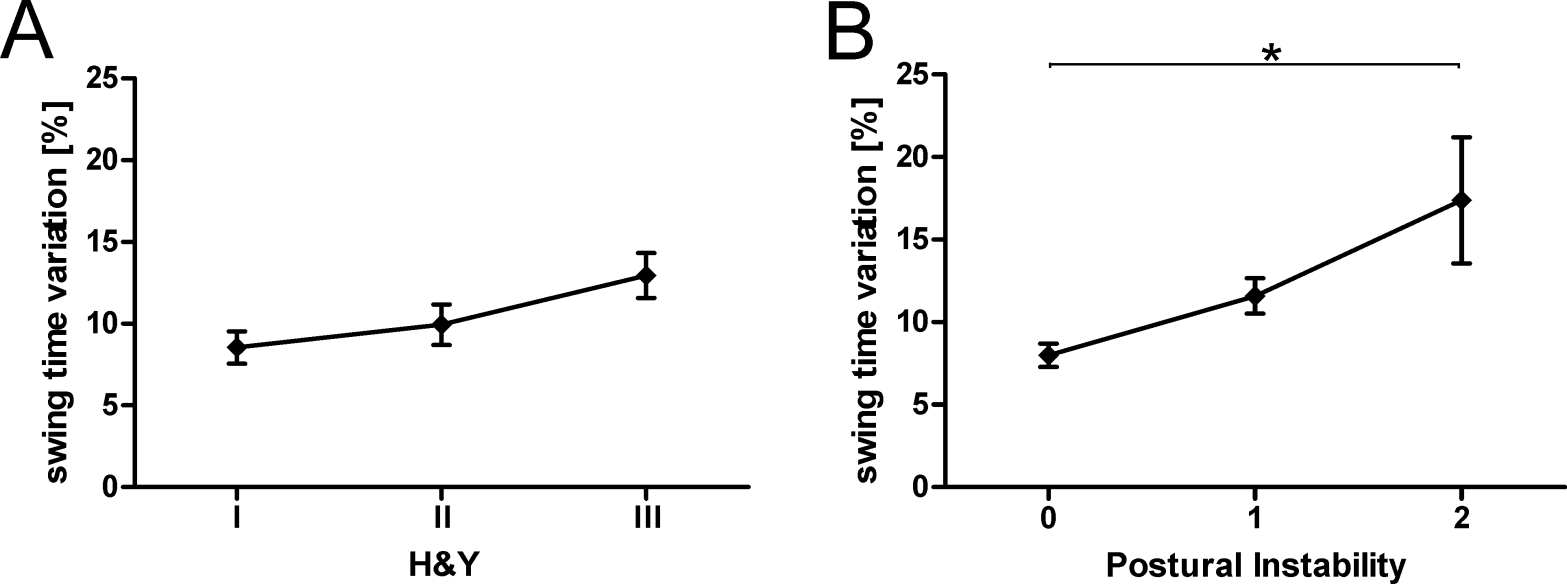
Swing time variation is increased in PD patients with increased postural instability. Swing time variation is unchanged when grouped according to H&Y disease stage (A). PD patients with higher postural instability, as identified by a rating of the single item "postural stability" of the UPDRS-III, show increased swing time variation (B). Group data are displayed as mean ± SEM. and compared using one-way ANOVA followed by Bonferroni’s post-hoc test. *p < 0.05.

Postural instability results in an increased risk for falls. In addition, non-harmonic gait and increased gait variation are highly prevalent in PD [29, 30]. An increased gait variation pattern, in particular stride time variation, has been associated with postural instability in PD [31]. Therefore, we analyzed whether gait variation was altered in the PD cohort. Interestingly, there was no group difference in gait variation (stride time, stride length, swing and stance phase time) between PD patients and controls (S3 Table). We then analyzed the impact of the disease stage according to H&Y on gait variation. Again, we did not detect significant differences, e.g., in swing time variation, when we compared different disease stages according to H&Y (Fig 4A). In the UPDRS-III, the patient’s performance in the pull test determines the clinician’s rating of postural stability. Intriguingly, we observed increased swing time variation in patients with increased postural instability compared to PD patients without postural instability (Fig 4B; One-way ANOVA followed by Bonferroni’s post-hoc test, F_(2, 159)_ = 13.1; p < 0.001). Thus, PD patients showing severe postural instability presented with increased swing time variation.

### Longitudinal assessment of gait parameters

**Fig 5:**
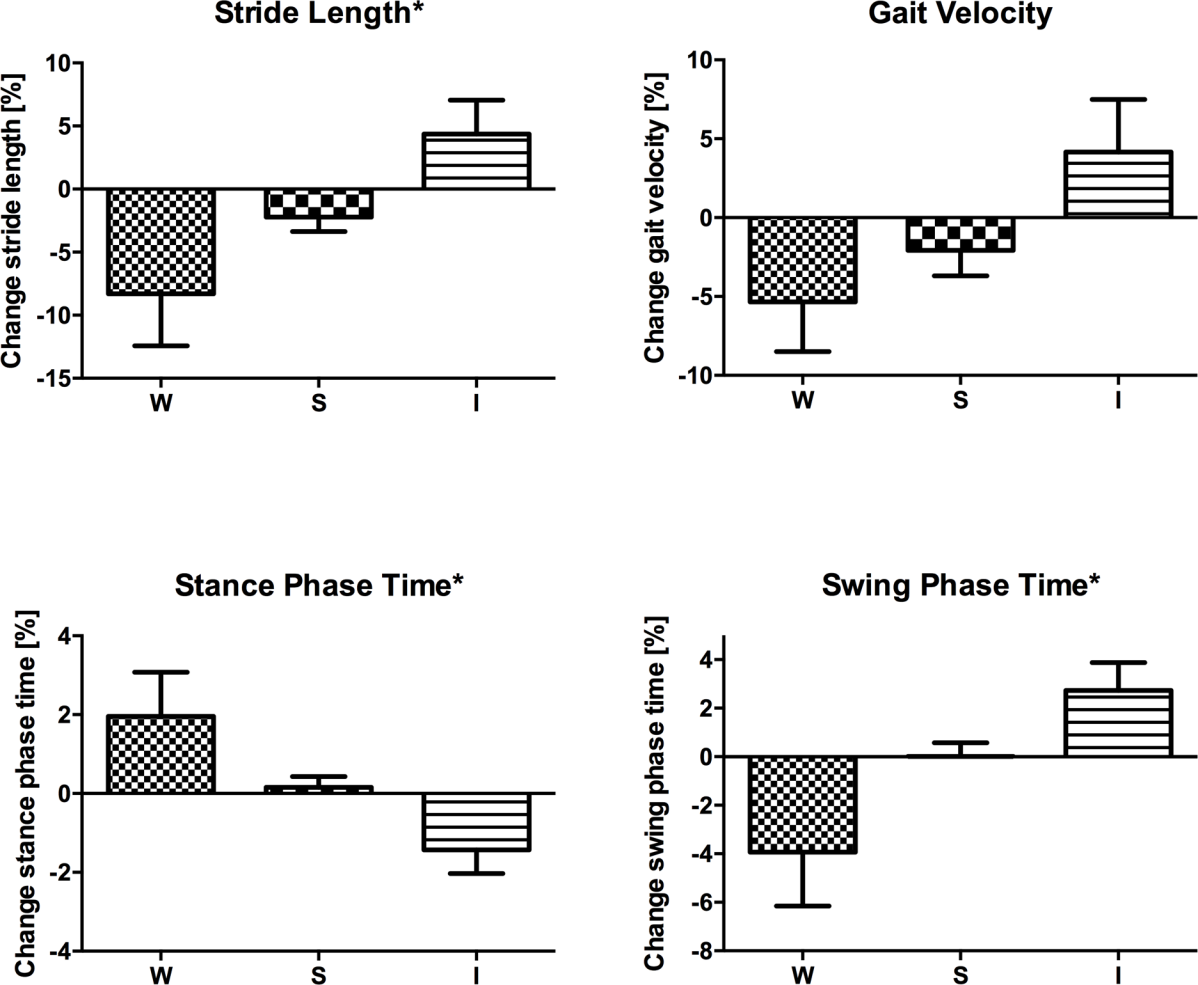
Longitudinal monitoring of individual gait parameters. Gait was rated and gait parameters were measured from PD patients at baseline and follow-up visit. According to the UPDRS-III single item "gait," patients improved (I), worsened (W), or were stable (S). The change in stride length (A), gait velocity (B), stance phase time (C), and swing phase time (D) are given in %. Data are displayed as mean ± SEM. and compared using one-way ANOVA followed by Bonferroni’s post-hoc test. *p < 0.01.

Next, we applied wearable sensor-based analysis to detect individual changes in gait parameters over time. 63 recurrent patients were examined clinically and gait parameters were recorded on both the first and the follow-up visit. Time between baseline and follow-up visit ranged between 1 and 45 months with a mean of 16.9 ± 1.5 months. We grouped the 63 PD patients according to the clinician’s rating of the UPDRS-III item “gait” into stable (S; no changes in gait item rating), improved or worsened (I/W: increase or worsening in gait item rating, respectively) groups irrespective of medication. Compared with the first visit, 13 patients improved, 37 were stable, and 13 worsened according to the UPDRS-III gait item at the follow-up visit. Our analysis revealed that in particular stride length reflected the changes of the clinician’s assessment of gait (One-way ANOVA followed by Bonferroni’s post-hoc test F_(2,61)_ = 5.7; P < 0.01; Fig 5A). Stride length did not differ significantly between the baseline and follow-up visit in patients who were stable in their gait. Intriguingly, patients who either improved or deteriorated in gait showed striking corresponding alterations in individual stride length. Patients with improved gait characteristics as rated by the clinician showed an increase in stride length by ~4.4%. In contrast, stride length was reduced by ~8.3% in patients with a deterioration in gait.

Changes in gait velocity over time, albeit not statistically significant, showed a tendency to reflect the clinician’s assessment of gait (F_(2,61)_ = 2.8; P = 0.07; Fig 5B). Gait velocity increased in patients with improved gait by 4.2%. Changes in stance phase time and swing phase time supported the clinical observation of the physicians (Stance phase time F_(2,61)_ = 6.4; P < 0.01; swing phase time F_(2,61)_ = 6.2; P < 0.01) as depicted in Fig 5C and 5D. For example, swing phase time was increased by about 2.7% in patients with improved gait rating in the UPDRS-III, and decreased by 3.9% in patients with worsened gait characteristics over time. Thus, using the present wearable sensor-based gait analysis, it was possible to perform individualized follow-up monitoring of spatial and temporal gait parameters during the disease course.

## Discussion

Translational neurotechnology is an emerging field with the potential to revolutionize the diagnosis of movement disorders. Here, we applied PD, the most prevalent basal ganglia disorder affecting gait, to wearable sensor-based gait analysis to objectively capture gait parameters in PD patients. All stride parameter measurements obtained with our wearable sensor-based gait analysis system were validated previously to a gold standard such as an optical motion capturing system [32] or instrumented walkway [21, 33]. In the latter two studies, gait parameters were validated in geriatric cohorts with more than 100 aged individuals. For example, the temporal parameter stride time as measured by the wearable sensor system had a mean absolute error of 9 ms and a standard deviation of 16 ms, whereas the spatial parameter stride length had a mean absolute error of 3.9 cm and a standard deviation of 4.7 cm both contributing to the technical variation of sensor-based gait analysis [32]. We now clinically validate the feasibility of sensor-based gait analysis technology in large scaled cross-sectional and longitudinal studies. This is one of the first study transferring technology readiness levels (TRL) from technical validation (TRL level 4) to system prototype demonstration and qualification in the operational environment of ambulatory healthcare (TRL level 7 and 8) [34], and thereby underlying supporting the clinical application of instrumented gait analysis in PD. For two centuries, detailed observation and clinical examination are the gold standard in neurology to assess PD associated gait alterations. Short steps and shuffling gait are prototypical features of PD-associated gait. By combining wearable sensors with advanced signal processing algorithms, we demonstrate the feasibility and application potential of wearable sensor-based gait analysis to objectively track group and individual PD-associated gait changes in cross-sectional and longitudinal approaches. Thus, our study strongly supports the implementation and application of wearable sensor-based gait analysis in the clinical routine.

One major goal of the present study was to investigate whether body worn sensor-based gait analysis detects clinically relevant gait pattern of PD patients that resemble and objectively complement the physician’s assessment. To this end, we recruited 190 PD patients and compared nine gait parameters with 101 age-matched controls in a cross-sectional study design. Wearable sensor-based gait analysis robustly tracks the most important features of PD gait, as illustrated by altered spatiotemporal gait parameters. Gait parameters differed significantly between controls and PD patients at moderate disease stage and/or higher levels of motor impairment.

Our findings confirm and extend previous reports on gait alterations in PD patients, in which only a limited number of PD patients were examined [16, 35–37]. One major advantage of the wearable sensor-based gait analysis system is that it does e.g. not require instrumented walkway and thus requiring a gait laboratory [38]. Although not the focus of this study, wearable sensors may also be applied to assess gait under free-living conditions, e.g. the patient’s home. Several studies, albeit with small patient numbers, addressed this important need [39–41]. These studies distinguished PD from controls based on gait parameters while walking outside and during turning. However, due to the small sample size, these studies were not designed to determine subtle differences in gait between early stage PD patients and controls.

Early diagnosis of PD can be challenging for neurologists [42]. Gait alterations in the early stages of PD are subtle and require a well-trained and experienced clinician to detect. Here, using wearable sensors, we detected reduced foot clearance as a single gait parameter to distinguish PD patients, irrespective of their disease stage, from controls. We showed previously that automated gait analysis via pattern recognition robustly distinguishes PD patients from controls and identified age as the most relevant disease independent confounder [17]. In the present study, we show that gait assessment by wearable sensor-based gait analysis provides an objective tool for characterization of PD gait parameters, additional assistance to diagnose PD, and individualized monitoring of gait during the course of the disease.

The UPDRS-III score is a standardized and internationally widely used clinical examination to assess motor function in PD. Whereas the UPDRS-III supports the physician in the clinical evaluation of PD patients, it suffers from inter- and intra-rater reliability and only partially reflects everyday life motor function [43, 44]. Here, we provide evidence for the unequivocal advantage of wearable sensor-based analysis in assessing motor function in PD by providing objective and subtle continuous measurements, thus complementing ordinal clinical scores such as the UPDRS-III.

Gait is a very important motor function in everyday life, and gait impairment is associated with reduced quality of life. Increased falls have been associated with increased stride-to-stride variation. We did not detect an overall difference in stride time and stride length variation in patients when analyzed for disease stage or motor impairment. Although we did not detect any differences when performing a group comparison analysis, swing time variation was significantly increased in PD patients with decreased postural stability, as determined by the clinician’s rating of the UPDRS-III item postural stability. Previous studies revealed that PD patients with greater postural instability showed increased gait variation [29, 31, 45]. This finding has important implications for medical and non-medical treatment strategies for PD patients showing increased stride variations because of an increased risk for falls and consequent future immobility [46]. In support of this, a recent report suggested that continuous gait monitoring detects higher step variability in PD patients with a positive history of falls compared to non-fallers [47]. Mobile wearable systems may have the potential to predict the patient’s risk of falling [48]. This may help facilitate the patient and physician to intervene and adjust treatment before falls occur. Indeed, our study supports the applicability of mobile sensors in clinical trials. Recently, wearable sensors were used in a clinical trial to detect changes in gait variability test as the primary outcome [49]. Step time variability during normal walking was reduced in PD patients on rivastigmine, suggesting that rivastigmine may exert a positive modulatory effect on gait stability [49].

However, stride variation assessed during the 4x10 meter walk in our study was higher than reported previously [50, 51]. First, this finding could be due to our experimental approach, since patients were asked to walk at their preferred speed. Previous studies have shown that different walking speeds or dual task conditions significantly alter stride variation [52, 53]. Second, longer gait sequences may reduce stride variation in particular when taking into account the acceleration phase [54, 55].

Another outstanding question of wearable sensor technology is whether it is capable to monitor gait during disease course. To meet the urgent need for long-term clinical and follow-up pharmacological studies at the patient level, it is necessary that the system captures individual gait differences longitudinally, i.e., to assess disease progression within patients or response to treatment. Thus, we re-examined 63 patients within a period of ~17 months, and subdivided them into stable, improved and worsened gait performer compared to their baseline visit. Changes in stride length coincided with the clinician’s rating of the UPDRS-III gait item. This finding was in line with the UPDRS-III rating system of the gait item, since it directed the clinician’s focus during visual analysis of stride length. These data demonstrate the feasibility of the wearable sensor based gait analysis to detect gait changes at the patient level. Our findings extend a previous study, which used a 7-m-long instrumented walkway to show that progression of gait abnormalities in PD is subtle [56]. Together with our recent study, which demonstrated that using wearable sensor technology enables the classification of PD patient [17], we provide the application of wearable sensor technology that goes beyond the mere detection of group differences. Although we show that body worn sensor-based gait analysis detects cross-sectional and longitudinal differences in gait parameter in PD patients, important demands for further clinical applicability such as detecting changes in gait in response to pharmacological intervention or long-term monitoring are at present lacking. Thus, future studies addressing the clinical benefits of sensor-technology will evaluate the sensitivity-to-change of the target parameters [6].

**Fig 6:**
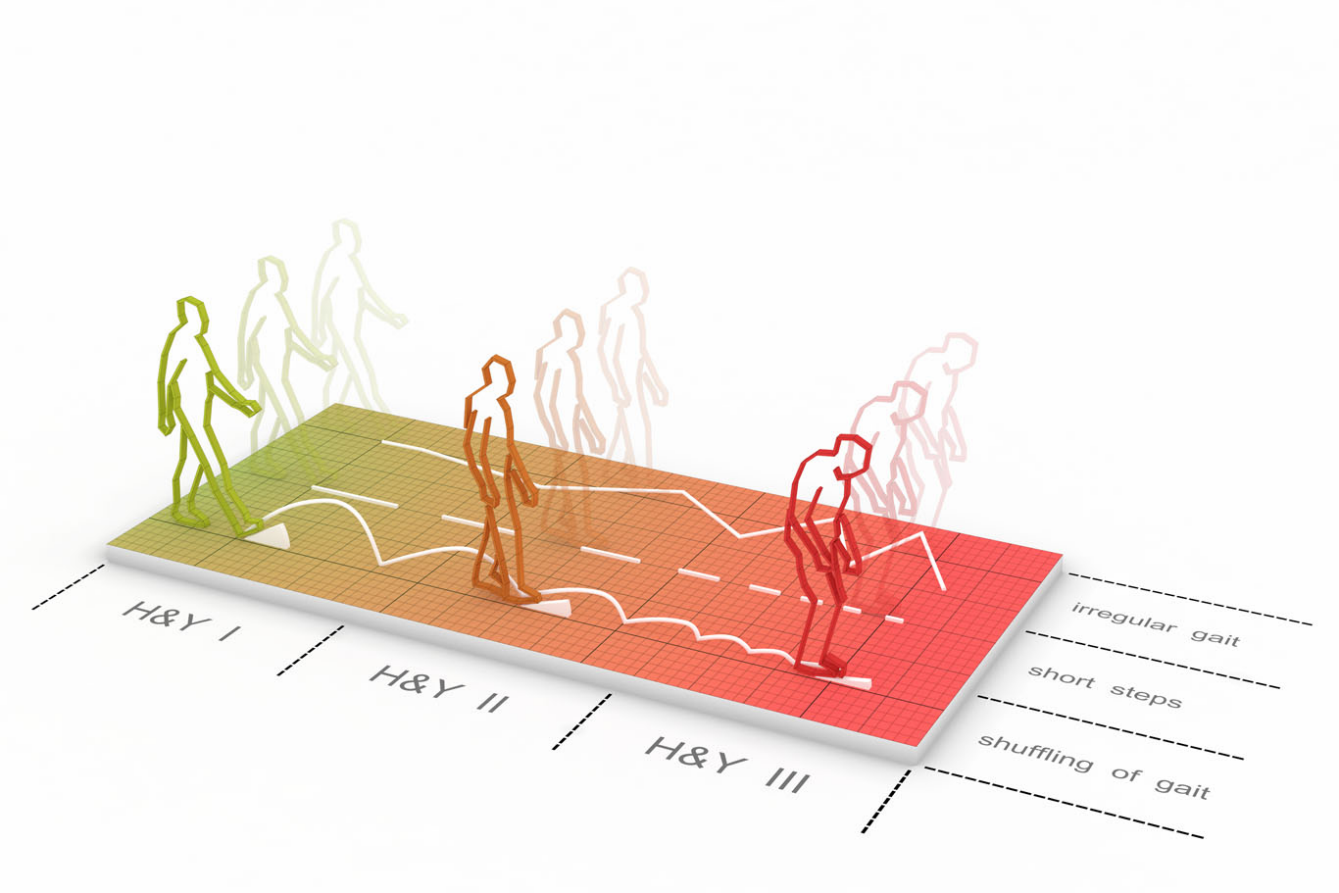
Scheme of measuring individual changes in gait parameters as measured by wearable sensors in PD during the course of the disease.

Mobile and wearable sensor technology is a rapidly evolving field with a high potential to transform health care and clinical research [6, 57, 58]. Here, we employed cross-sectional analysis and individualized longitudinal follow-ups to assess PD gait signatures via wearable sensor-based gait analysis. Our findings show that this method provides objective and quantitative outcomes of distinct gait parameters (Fig 6), which have the potential to serve as a kinetic biomarker. Wearable sensor-based gait analysis advances the study of PD in terms of clinical characterization, longitudinal disease progression, and objective and quantitative evaluation of interventions. From the patient’s point of view, these parameters may be used as valid and reliable biofeedback in everyday life. PD is an ideal proof-of-concept disease for instrumented gait analysis, since gait impairment is a frequent symptom in numerous disorders, including musculoskeletal conditions and metabolic syndromes. By adapting devices like wearable sensor-based gait analysis to capture disease state, translational neurotechnologies as demonstrated here can meet both the physician’s, patient’s and caregiver’s needs.

## Supporting information

S1 Fig Placement of the sensor and overview of calculated gait parameters. The sensor units consisting of a tri-axial accelerometer and a tri-axial gyroscope were laterally attached to the lateral side of each shoe (A). A robust, template-based stride detection program allowed calculation of distinct features of the gait cycle like stride length/time, stance phase and swing phase times, heel strike (HS) and toe off angles (TO) as well as foot clearance (FC) (B).
(TIF)

S2 Fig Visualization of data processing to calculate stride parameters. Abbreviations: IMU: inertial measurement unit; msDTW: multi-dimensional dynamic time warping.
(TIFF)

S1 Table Summary and definition of gait parameters. Abbreviations: m = meter; ms = millisecond; spm = steps per minute; cm = centimeter; CV = coefficient of variation.
(DOCX)

S2 Table Gait parameters of the 4x10 meter walk *without* initiation steps. Values reported as mean ± SEM. Abbreviation: w/o = without.
(DOCX)

S3 Table Gait variability during the 4x10 meter walk with and without initiation steps. Values reported as mean ± SEM. Abbreviation: w/o = without.
(DOCX)
