# Supplementary figures and images for: Wearable sensors objectively measure gait parameters in Parkinson’s disease
Source: PLoS One. 2017 Oct 11;12(10):e0183989. doi: 10.1371/journal.pone.0183989 (PMC5636070; doi:10.1371/journal.pone.0183989)

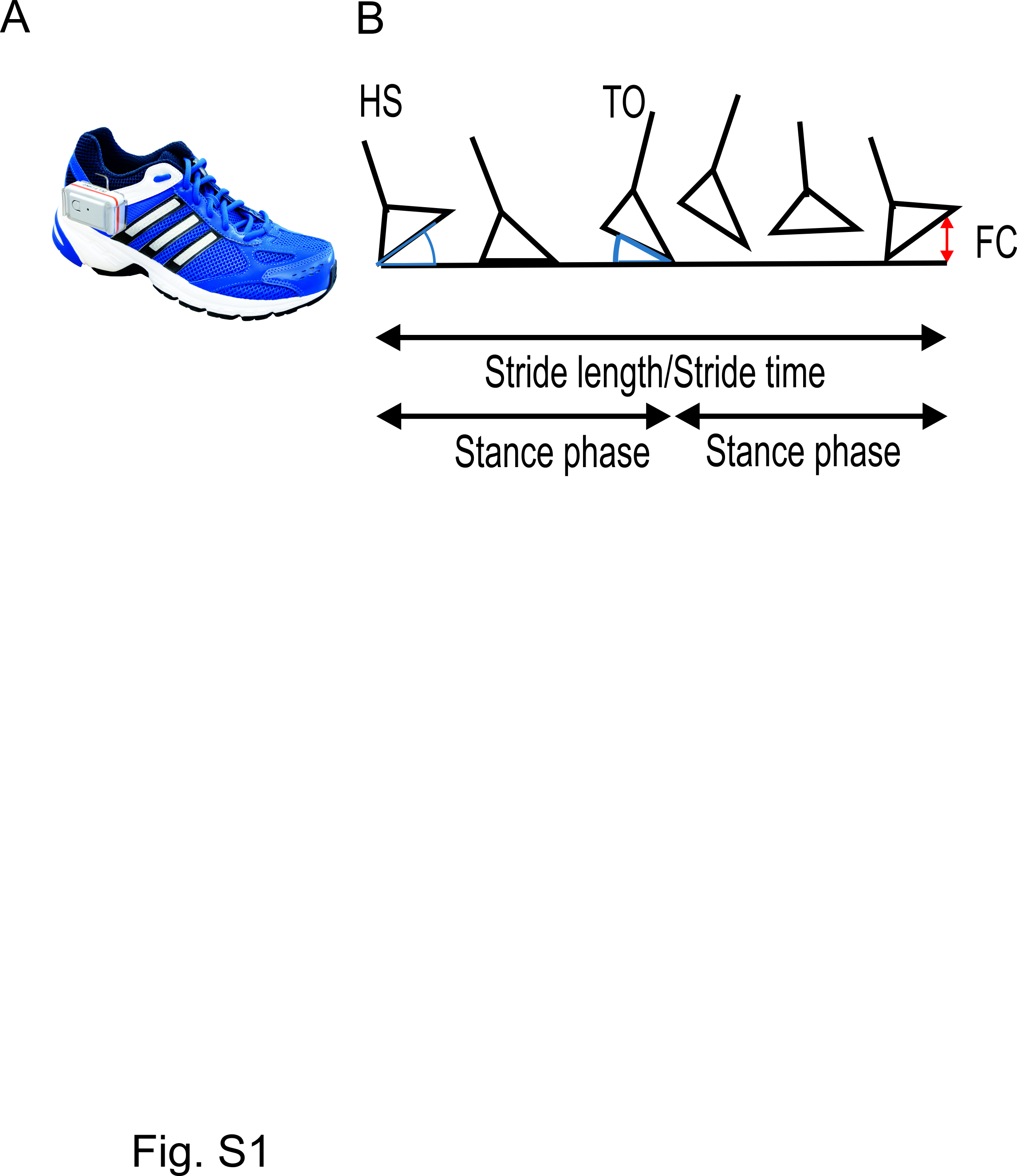

Supplement: S1 Fig — The sensor units consisting of a tri-axial accelerometer and a tri-axial gyroscope were laterally attached to the lateral side of each shoe (A). A robust, template-based stride detection program allowed calculation of distinct features of the gait cycle like stride length/time, stance phase and swing phase times, heel strike (HS) and toe off angles (TO) as well as foot clearance (FC) (B). (TIF) [file pone.0183989.s001.tif]

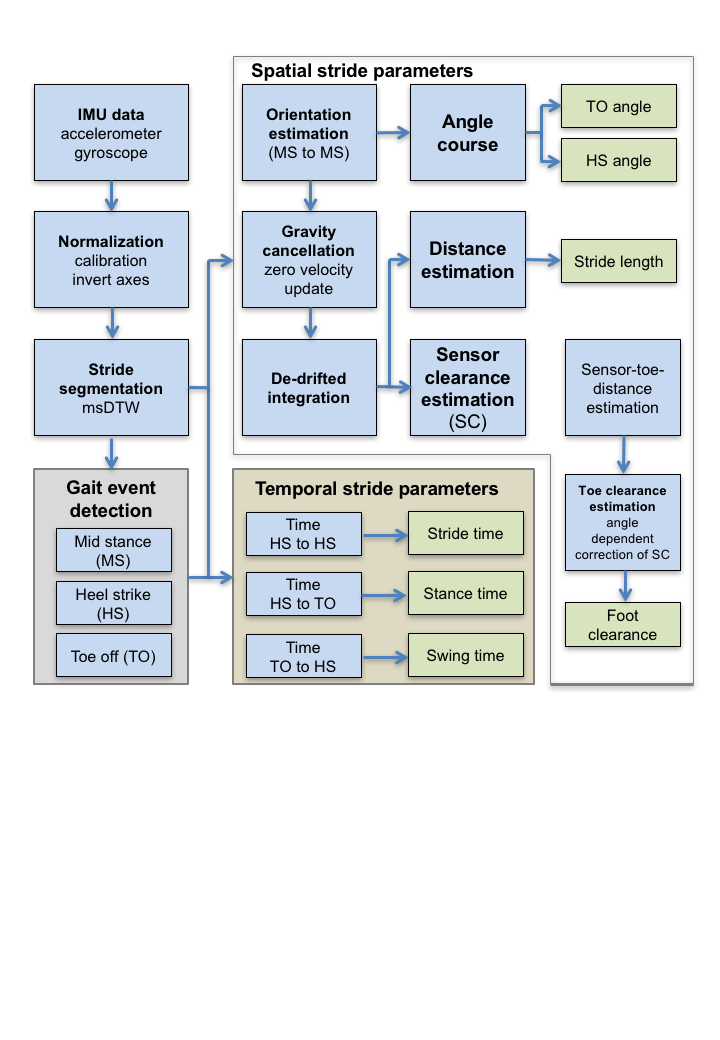

Supplement: S2 Fig — Abbreviations: IMU: inertial measurement unit; msDTW: multi-dimensional dynamic time warping. (TIFF) [file pone.0183989.s002.tiff]
