# Supplementary material for: Wearable sensors objectively measure gait parameters in Parkinson’s disease
Source: PLoS One. 2017 Oct 11;12(10):e0183989. doi: 10.1371/journal.pone.0183989 (PMC5636070; doi:10.1371/journal.pone.0183989)
Supplement: S1 Table — Abbreviations: m = meter; ms = millisecond; spm = steps per minute; cm = centimeter; CV = coefficient of variation. (DOCX) [file pone.0183989.s003.docx]

| **Gait Parameter** | **Definition** |
| --- | --- |
| Stride length [m] | Distance between two sequential points of midstance contacts with the same foot |
| Stride time [s] | Duration of one gait cycle |
| Gait velocity [m/s] | Walking speed in a designated direction |
| Cadence [spm] | Step rate per minute |
| Stance phase time [%] | Period in gait when the foot is in contact with the floor |
| Swing phase time [%] | Period in gait cycle when the foot is *not* in contact with the floor |
| Foot clearance [cm] | Maximum toe height during swing phase |
| Heel-strike angle [°] | Angle between foot and ground in the sagittal plane at heel-strike |
| Toe-off angle [°] | Angle between foot and ground in the sagittal plane at toe-off |
| Gait variation | Magnitude of alterations in individual gait parameters during walking, also defined by the CV: ratio of standard deviation to the mean) |

**S1 Table: Summary and definition of gait parameters**

Abbreviations: m=meter; ms=millisecond; spm=steps per minute; cm=centimeter; CV=coefficient of variation
