# Supplementary material for: Wearable sensors objectively measure gait parameters in Parkinson’s disease
Source: PLoS One. 2017 Oct 11;12(10):e0183989. doi: 10.1371/journal.pone.0183989 (PMC5636070; doi:10.1371/journal.pone.0183989)
Supplement: S2 Table — Values reported as mean ± SEM. Abbreviation: w/o = without. (DOCX) [file pone.0183989.s004.docx]

**S2 Table: Gait parameters of the 4x10 meter walk *without* initiation steps**

| **Variables** | **PD** | **Controls** | **P** |
| --- | --- | --- | --- |
| **Stride length [m]** | 1.13 ± 0.01 | 1.22 ± 0.01 | **< .001** |
| **Norm. stride length** | 0.69 ± 0.02 | 0.74 ± 0.01 | **< .001** |
| **Stride time [ms]** | 1.08 ± 0.01 | 1.04 ± 0.01 | **< .001** |
| **Gait velocity [m/s]** | 1.11 ± 0.01 | 1.23 ± 0.02 | **< .001** |
| **Cadence [spm]** | 56.0 ± 0.4 | 58.6 ± 0.7 | **< .001** |
| **Stance phase time [%]** | 64.7 ± 0.2 | 63.9 ± 0.2 | **< .001** |
| **Swing phase time [%]** | 35.3 ± 0.2 | 36.1 ± 0.2 | **< .001** |
| **Foot clearance [cm]** | 10.7 ± 0.3 | 14.6 ± 0.5 | **< .001** |
| **Heel-strike angle [°]** | 19.7 ± 0.7 | 23.6 ± 0.8 | **< .01** |
| **Toe-off angle [°]** | -55.3 ± 0.7 | -56.5 ± 0.8 | > .05 |

Values presented as mean ± SEM. Significance was determined by *t-test*.

Abbreviation: Norm. = normalized
