# Supplementary material for: Wearable sensors objectively measure gait parameters in Parkinson’s disease
Source: PLoS One. 2017 Oct 11;12(10):e0183989. doi: 10.1371/journal.pone.0183989 (PMC5636070; doi:10.1371/journal.pone.0183989)
Supplement: S3 Table — Values reported as mean ± SEM. Abbreviation: w/o = without. (DOCX) [file pone.0183989.s005.docx]

**S3 Table: Gait variability during the 4x10 meter walk with and without initiation steps**

| **Variables** | **PD** | | **Controls** | | **P** |
| --- | --- | --- | --- | --- | --- |
|  | **with Initiation** | **w/o**  **Initiation** | **with Initiation** | **w/o**  **Initiation** |  |
| Stride length CV (%) | 15·8 ± 5·7 | 8·3 ± 4·3 | 15·7 ± 7·6 | 9·1 ± 4·2 | > 0·05 |
| Stride time CV (%) | 7·0 ± 4·3 | 5·6 ± 5·1 | 7·8 ± 5·4 | 6·1 ± 5·3 | > 0·05 |
| Stance phase CV (%) | 8·5 ± 5·2 | 6·3 ± 5·7 | 9·3 ± 6·0 | 7·3 ± 6·3 | > 0·05 |
| Swing phase CV (%) | 10·5 ± 9·7 | 8·9 ± 10·6 | 11·1 ± 12·5 | 9·2 ± 10·1 | > 0·05 |

Values reported as mean ± SEM. Abbreviation: w/o=without
